# Optimizing RNA extraction methods for high-throughput transcriptome sequencing of formalin-fixed paraffin-embedded cardiac tissue specimens

**DOI:** 10.1371/journal.pone.0315098

**Published:** 2024-12-26

**Authors:** Nkechi Martina Odogwu, Jin Sung Jang, Sabrina Albertson, Clinton Hagen, Boyd Rasmussen, Oommen Saji, Timothy J. Nelson, the Wanek Program Preclinical Pipeline

**Affiliations:** 1 Program for Hypoplastic Left Heart Syndrome, Mayo Clinic Rochester, Rochester, Minnesota, United States of America; 2 Genome Analysis Core, Medical Genome Facility, Center for Individualized Medicine, Mayo Clinic Rochester, Rochester, Minnesota, United States of America; 3 Department of Laboratory Medicine and Pathology, Mayo Clinic Rochester, Rochester, Minnesota, United States of America; 4 Division of Cardiovascular Medicine, General Internal Medicine, Mayo Clinic Rochester, Rochester, Minnesota, United States of America; 5 Molecular Pharmacology and Experimental Therapeutics, Mayo Clinic Rochester, Rochester, Minnesota, United States of America; 6 Center for Regenerative Medicine, Mayo Clinic Rochester, Rochester, Minnesota, United States of America; Universidad de Jaen, SPAIN

## Abstract

Archived FFPE cardiac tissue specimens are valuable for molecular studies aimed at identifying biomarkers linked to mortality in cardiovascular disease. Establishing a reliable and reproducible RNA extraction method is critical for generating high-quality transcriptome sequences for molecular assays. Here, the efficiency of four RNA extraction methods: Qiagen AllPrep DNA/RNA method (Method QP); Qiagen AllPrep DNA/RNA method with protocol modification on the ethanol wash step after deparaffinization (Method QE); CELLDATA RNA extraction (Method BP) and CELLDATA RNA extraction with protocol modifications on the lysis step (Method BL) was compared on 23 matching FFPE cardiac tissue specimens (n = 92).In comparing RNA quality metrics across FFPE RNA extract, nucleic acids extracted deploying Method QE and QP produced the highest RNA yield. However, Method QE outperformed Method QP as more extract from Method QE had DV 200 values above 30%. Both method BL and BP produced similar range of RNA purity and yield but more extract from Method BL had DV 200 values above 30% compared to Method BP. When accessing distribution value, Method BL outperformed Methods BP, QE, and QP as more extracts from Method BL had DV 200 values above 30% compared to other methods (P_DV200_<0.001; Kruskal-Wallis). Method QE outperformed other methods in terms of RNA yield. RNA extracts from Method QE, characterized by high RNA yield, achieved sequencing results comparable to those from Method BL, characterized by high DV200 values. Our findings reveal that optimizing protocols can yield higher-quality RNA, facilitating the exploration of more disease conditions with high-resolution transcriptome profiling.

## Introduction

Cardiovascular diseases (CVDs) are the most common and fatal disease in the United States [1]. In 2008, approximately 17.9 million people died from CVDs, representing 32% of global deaths [2]. Based on numerous lines of research, most CVDs are believed to be multifactorial genetic conditions, involving multiple genes or environmental factors [3, 4]. The current standard for studying certain genetically related CVDs is establishing preclinical animal models recapitulating actual CVDs in humans and deciphering gene expression profiles across cardiac tissues and liquid biopsies (whole blood, plasma, serum, buffy coat, isolated blood cells) via robust sequencing technology [5, 6]. Biological processes associated with diseases can be delineated by pinpointing transcriptomes and their changes in expression at the RNA level [7, 8]. Worth mentioning is the fact that in any transcriptome profiling study, minimal RNA degradation is paramount to reliable downstream analysis. In the context of clinical samples especially in cardiovascular research, obtaining samples with intact RNA can be challenging with the scenario of samples remaining on the bench at room temperature while the cardiologist tends to the patient.

FFPE-preserved samples are a great source of treasure as interesting tissue samples are archived for future investigation however, clinically relevant questions remain, such as what assay method across several commercially available kits can provide quality RNA extract, what quality metrics or analytical variables can accurately predict the successful performance of FFPE-derived RNA on molecular platforms, and how accurate are the results from the derived data? Fixation of tissues causes modifications of biomolecules such as cross-linkage of nucleic acids with proteins, RNA fragmentation, and covalent modifications of both DNA and RNA, making it challenging to extract nucleic acids of high quality from FFPE tissues [9, 10]. Aside from RNA fragmentation, the quality of RNA is affected by other parameters such as time from sample retrieval to fixation, duration, and conditions of fixation, the paraffin embedding procedure, and sample storage, which can impact sequence outcome [11, 12]. Therefore, it is important to truly assess the accuracy and reproducibility of various protocols. As an illustration, in an Equine model, Boos and others demonstrated that tissue-specific adaptation may impact the sensitivity of the molecular technique, therefore optimizing RNA methods beyond the recommended commercial manufacturer’s methods becomes extremely important [13]. Carithers et al. (2021), work also demonstrated differences in RNase activity across various tissue types [14]. Currently, available commercial kits for tissue specimens may not consider these differences in RNAse activity across various tissue types therefore optimizing or adding steps to improve the quality of RNA extract for better transcriptome sequence outcomes remains crucial [15]. Here we hypothesize that additional steps beyond the manufacturer’s protocol could improve RNA yield and integrity of FFPE-derived samples and subsequently improve sequence outcomes. Furthermore, RNA quality metrics such as RNA yield, RNA purity (A260/230 and A260/280), RIN integrity, and RNA fragment length (DV200) are also hypothesized to impact sequencing outcomes [16], however, these variables have not been extensively studied in FFPE cardiac tissues.

As transcriptome analysis becomes more important in routine patient diagnosis and as evidence builds that certain FFPE-processing parameters, such as prolonged formalin fixation of 48 to 72 hours, and prolonged time of storage (more than one year) may compromise molecular analysis and sequence outcome [17, 18], researchers may wish to explore ways to improve practices by optimizing and evaluating multiple protocols for better transcriptomic sequencing outcomes. Here we describe optimized methods of RNA isolation from FFPE-cardiac tissues in a non-human primate (*Macaca mulatta*) model of right ventricular pressure overload, we discuss the respective performance of the different RNA extraction protocols on gene expression detection and pinpointed suitable reproducible methods that consistently yielded high-quality transcriptome sequences, enabling the study of differentially expressed genes.

## Methods

### Animal welfare

This study was performed in compliance with the Animal Welfare Act (United States Code, Title 7, Chapter 54) and the United States Department of Agriculture Animal Welfare Regulations (Code of Federal Regulations, Title 9, Chapter 1, Subchapter A, Parts 1–4). The protocol was reviewed and approved by the WNPRC (Wisconsin National Primate Research Center) Institutional Animal Care and Use Committee (IACUC). All experiments were performed in compliance with the ARRIVE guidelines as previously described [19]. Non-human primates of *Macaca mulatta*, a breed of Indian origin, were born in-house at the Wisconsin National Primate Research Center (WNPRC) or sourced from a WNPRC-approved vendor in accordance with WNPRC requirements. Study animals were housed in standard caging according to WNPRC institutional guidelines and in compliance with the space requirements of the Guide for the Care and Use of Laboratory Animals and Animal Welfare Regulations. A comprehensive environmental enrichment program was put in place to ensure the psychological well-being of the nonhuman primates housed in this facility. Temperature and humidity are monitored daily by animal care personnel. Temperature is maintained between 65-75ºF and humidity between 30–70%. Study animals were fed a standard chow formulated for nonhuman primates. Fresh produce and treats were provided daily, and foraging devices were provided at least weekly following WNPRC procedures. Water was provided ad libitum. Euthanasia by exsanguination under deep anesthesia was performed at the end of the study. Animals were deeply anesthetized with ketamine followed by pentobarbital and the thoracic cavity was surgically opened to allow cannulation of the left auricle and transection of the right auricle. The descending aorta was clamped, and physiological heparinized saline was perfused followed by 4% paraformaldehyde. Death was verified by visual observation of cessation of a heartbeat. Cardiac tissue samples were collected by a certified pathologist and embedded in paraffin blocks. Samples were archived at room temperature (RT) for over 1 year.

### Assessment of RNA extraction protocols

The following kits and modifications were tested for evaluation of the most effective RNA isolation method: the AllPrep DNA/RNA FFPE kit (catalog #80234, Qiagen, Hilden, Germany) (**Method QP**), AllPrep DNA/RNA FFPE kit (catalog #80234, Qiagen, Hilden, Germany), with protocol modification on the ethanol wash step after deparaffinization (**Method QE**), CELLDATA RNAstorm 2.0 FFPE RNA Extraction kit (catalog **#**CD506 Biotium—USA) (**Method BP**) and CELLDATA RNAstorm 2.0 FFPE RNA Extraction Kit (catalog **#**CD506 Biotium—USA) with protocol modifications on the lysis step **(Method BL)** (Table 1).

**Table 1:** Overview of FFPE cardiac tissue RNA extraction methods.

Characteristics	Method QP	Method QE	Method BP	Method BL
Kit name	AllPrep RNA FFPE kit	AllPrep RNA FFPE kit with protocol modification on the ethanol wash steps after deparaffinization	CELLDATA RNAstorm 2.0 FFPE RNA Extraction Kit	CELLDATA RNAstorm 2.0 FFPE RNA Extraction Kit with protocol modification on lysis step
Supplier	Qiagen	Qiagen	Biotium	Biotium
Level of automation	Manual	Manual	Manual	Manual
Deparaffinization	Xylene	Xylene	Deparaffinization using included reagent	Deparaffinization using included reagent
Protocol Modification	No- Only 1 ethanol wash step in (96–100%) after deparaffinization with xylene	Yes- 3 ethanol wash steps (96–100%) twice and in 70% ethanol with 2 mins centrifugation at each wash step	No- Mixing RNAstorm FFPE Lysis Buffer with RNAstorm^™^ FFPE Protease and incubating at 72°C for 2 hours.	Yes- Mixing RNA storm FFPE Lysis Buffer with RNAstorm^™^ FFPE Protease and incubating at 72°C for 24 hours.
Hands-on time from section preparation to elution	2 h 00 min	2 h 00 min	2 h 00 min	2 h 00 min
Incubation and centrifugation	1 h 10 min	1 h 30 min	3 h 30 min	25 h 30min
Total turnaround time	3 h 10 min	3 h 30 min	5 h 30 min	27 h 46 min
DNase treatment	Included	Included	Included	Included
Eluate volume	20 μL	20 μL	50 μL	50 μL

### RNA extraction

**Fig 1:**
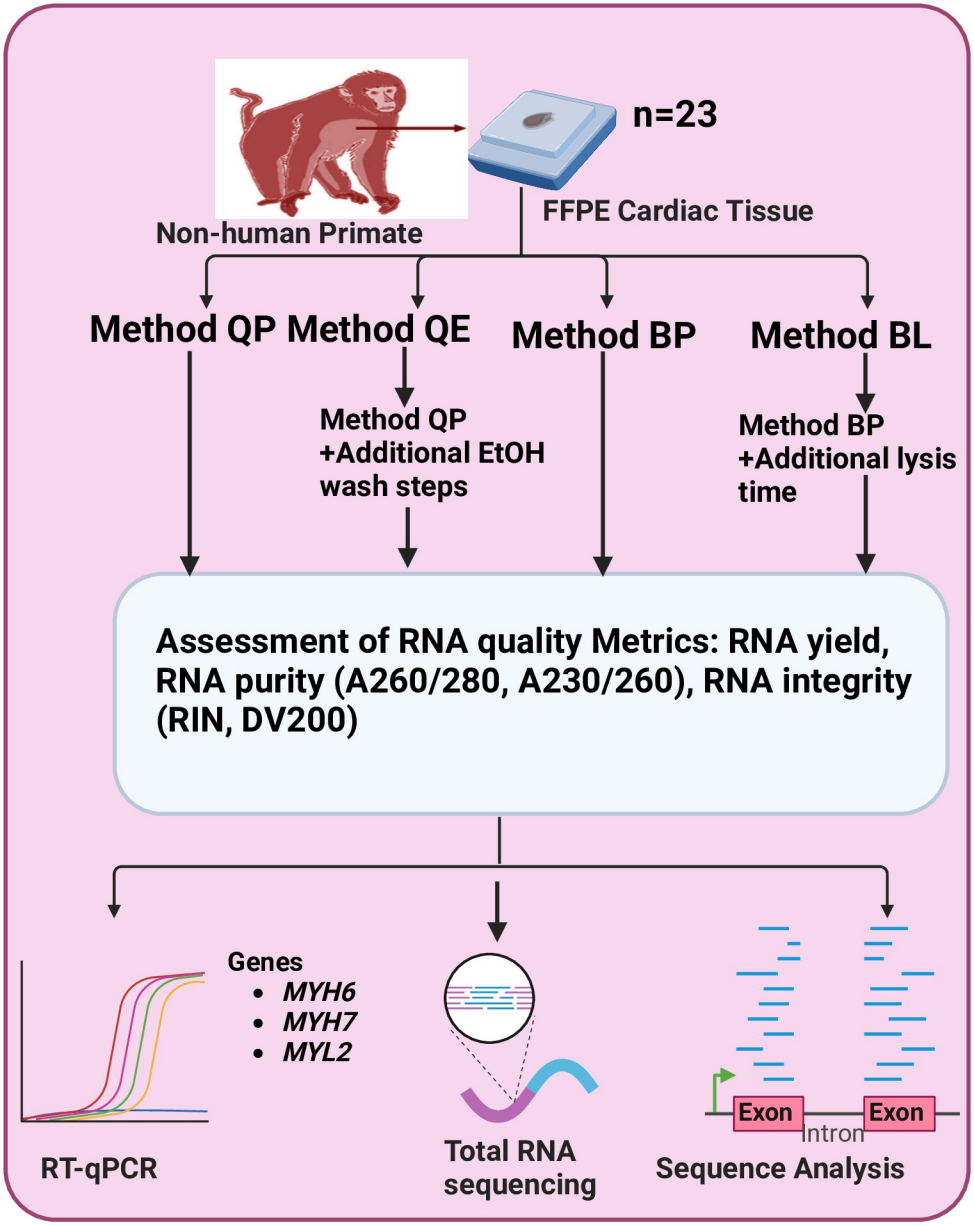
Overview of experiment design.

All isolation procedures were performed under standard laboratory conditions. The overview of experimental design is described in Fig 1. FFPE tissues were processed, and RNA was extracted. Genomic DNA was removed during RNA extraction for all methods. Total RNA concentration was qualified by Nanodrop (Nanodrop Technologies, Wilmington, DE) and the Agilent Fragment Analyzer (Santa Clara, CA). RNA quantity was determined using Qubit fluorometry (ThermoFisher Scientific, Waltham, MA). The RNA purity was determined by measuring the 260/280 and 260/230 nm absorbance ratios, using NanoDrop ND-1000 spectrophotometer. The distribution value 200 (DV200) quality metric which describes the percentage of RNA fragments longer than 200 nucleotides was determined using Agilent fragment analyzer, which separates DNA, RNA, and protein samples based on electrophoretic and microfluidic characteristics. DV200 is routinely used as an assessment standard for RNA quality because of its high correlation with the library yield of FFPE samples.

### Cardiac gene amplification (Quantitative Real-time Polymerase chain reaction, RT-PCR)

DNase-treated total RNA was reverse transcribed and amplified by real-time quantitative PCR on a QuantStudio 7 Pro System (Applied Biosystems). The relative mRNA expression of genes was examined using quantitative PCR with gene-specific primer sets (IDT, USA). Primers were synthesized with the Integrated DNA Technologies Primer Quest Tool. Amplification of the following genes: MYL2, MYH6, and MYH7 was done using the QuantiTect Reverse Transcription Kit (QIAGEN GmbH, Hilden, Germany) Thermocycling condition was set as follows: 95 °C for 5 min, 35 cycles of 98 °C for 20 s, 55 °C for 19 s, and 72 °C for 60 s, a final 72 °C extension for 5 min and hold at 4 °C. Each sample was run in duplicate and analyzed using the QuantStudio 7 Pro System design and analysis software. Primer sequences of each gene are provided in S1 Table.

### RNA-Seq library construction and data analysis

Sequencing was performed in collaboration with the Mayo Clinic Genome Analysis Core. Total RNA sequencing was performed using the Illumina Stranded Total RNA-Seq Ligation kit (Illumina, San Diego, CA) on RNA samples obtained from non-human primates. Each 1 μg of FFPE-derived RNA was treated with DNaseI (Zymo, USA) to remove single-stranded DNA. This was followed by an RNA XP bead (Beckman Coulter, USA) clean-up step for RNA purification. Before library construction, the RNA was quantified using Nanodrop (Thermo, USA) and 100ng was taken for rRNA removal using the Ribo-Zero Plus rRNA Depletion Kit (Illumina, USA). Briefly, the 100ng of total RNA was hybridized with single-strand DNA probes, and RNase H digestion was performed. DNaseI was used to remove the excessive single-strand DNA probes after RNase H digestion. The depleted RNAs were then enriched with 1.8 times amounts of Ampure RNA Clean XP beads and eluted with 10 μl of nuclease-free water. The RNA-Seq library was generated without fragmenting the RNA. The constructed libraries were quantified using the TapeStation 4200 system (Agilent, USA) and Qubit dsDNA BR Assay kits (Thermo Fisher, USA). The libraries were sequenced following Illumina’s standard protocol for the Illumina NextSeq 2000. The NextSeq P2 flow cells were sequenced as 101 bp paired-end reads using NextSeq 1000/2000 Control Software Suite v1.5.0 and RTA3. All libraries were sequenced 101 bp paired end reads on Illumina NovaSeq 6000. Approximately 56 to 87 million (M) pairs of total reads were generated from each library. FASTQ files were uploaded into Partek Flow software (Partek Inc., USA) for primary QC. The STAR (2.7.8a) aligner was used to align reads to the Macaca mulatta (Mmul_10) reference genome. The generated BAM files were quantified using the Partek E/M algorithm [20] (Xing et al, 2006) by Ensembl annotations (Ensembl Transcripts release 110) and then normalized using the Median of ratios method [21]. The BAM files were also used for rRNA quantification, calculated using the percentage of the total mapped reads using the Partek E/M algorithm. Normalized expression values (log2-transformed + 1) were used for sample correlation analysis. Sequence mapping to the genome and the transcriptome was visualized in Integrative Genomics Viewer (IGV, Broad Institute) [22].

### Statistical analysis

All continuous variables are presented as median (IQR) and categorical data are presented as count (%). Statistical testing of continuous variables between dependent group distributions was done using the Wilcoxon rank sum test (W) to account for the paired nature of these data. The Kruskal-Wallis rank sum test (KW) was used for continuous variables to test between independent group distributions. Categorical variables were tested using the Chi-square test. No adjustments were made for multiple testing, the results presented represent all results and are not limited to statistically significant results with Type I error rate of 0.05. Analyses were conducted using SAS/STAT software, Version 9.4 for Windows Server, and R statistical software [23]. For all groups, an asterisk (*) denotes a significant difference between groups where (*p<0.05, ***p<0.005, ****p<0.001).

## Results

### Comparison of RNA extraction methods from FFPE cardiac tissues

**Fig 2:**
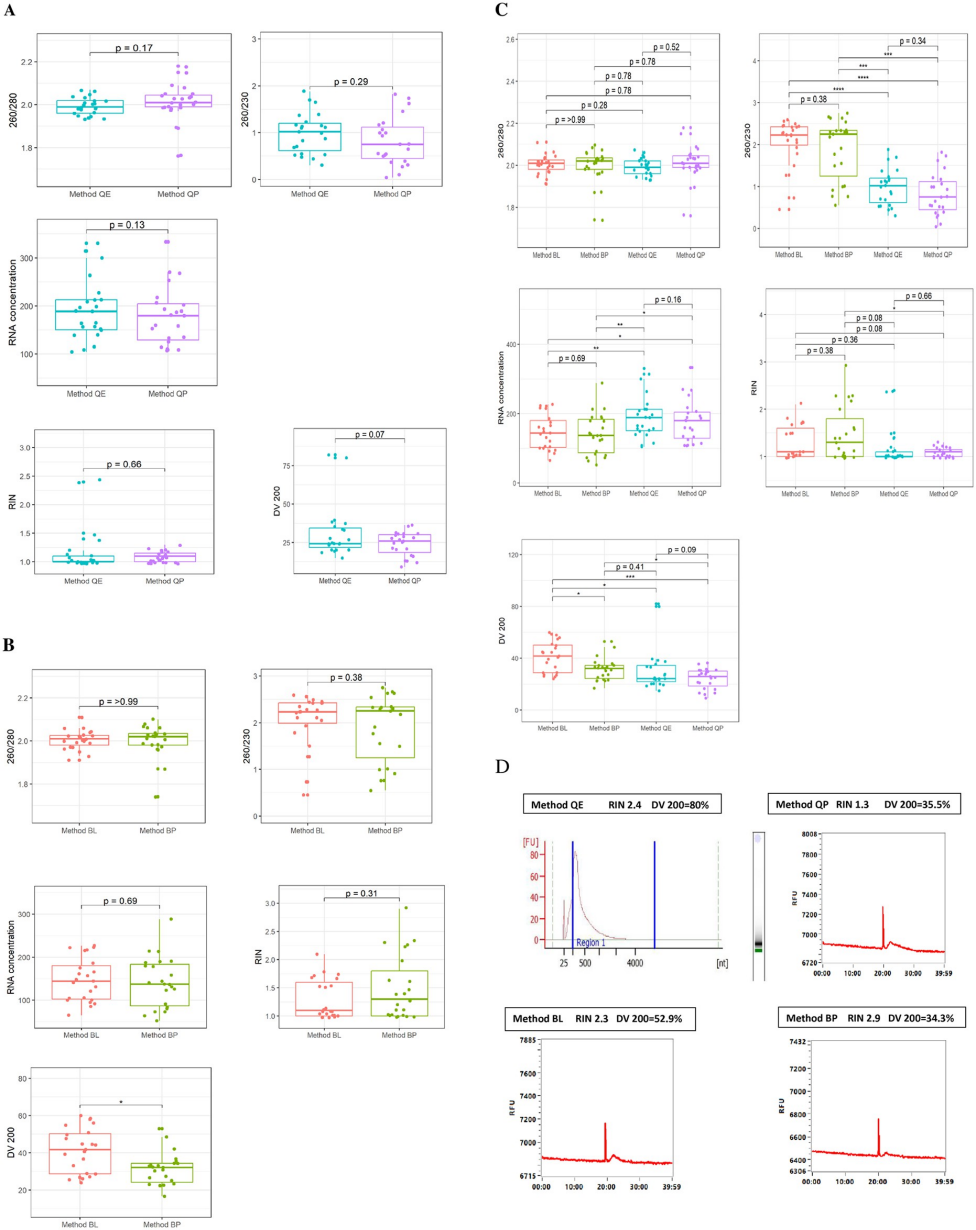
Assessment of RNA quality metrics in cardiac tissue FFPE derived RNA extract. **(A**) Box and whisker plots (min to max) of RNA concentration and RNA purity assessment of 260/230 and A260/280 absorbance ratio based on absorption spectra in RNA samples extracted using Method QP versus Method QE **(B)** Box and whisker plots (min to max) of RNA concentration and RNA purity assessment of 260/230 and A260/280 absorbance ratio based on absorption spectra in RNA samples extracted using Method BL versus Method BP (**C)** Comparison across four methods of extraction, Box and whisker plots (min to max) of RNA concentration, RNA purity assessment of 260/230 and A260/280 absorbance ratio across method BL and BP vs Method QE and Method QP **(D)** Electrophoregram/RNA fragment length across the four methods. For all groups, the asterisk (*) denotes a significant difference between groups, Paired Wilcoxon Signed Rank Test (*p<0.05, ***p<0.005, ****p<0.001).

Comparing method QP and QE, we found no significant difference in RNA yield, RNA purity DV 200, and RIN value except that more samples had DV 200 above 30% with our optimization step (Method QE) compared to Method QP (8 of 23 vs 6 of 23 samples) (Table 2) (Fig 2A) shows a comparison of RNA yields, purity(A260/280), RIN, and DV 200 values obtained from samples extracted using the four methods.

**Table 2:** Comparison of RNA quality metrics across RNA extraction from FFPE cardiac tissue samples.

	Method BL (N = 23)	Method BP (N = 23)	Method QE (N = 23)	Method QP (N = 23)	p value
**260/280**					0.265^1^
Median (Q1, Q3)	2.01 (1.98, 2.02)	2.02 (1.98, 2.04)	1.99 (1.96, 2.02)	2.01 (1.99, 2.04)	
**260/230**					< 0.001^1^
Median (Q1, Q3)	2.23 (1.99, 2.42)	2.25 (1.25, 2.33)	1.02 (0.61, 1.20)	0.75 (0.44, 1.11)	
**RNA. Concentration**					0.024^1^
Median (Q1, Q3)	144.36 (102.60, 180.37)	137.37 (87.34, 183.47)	188.66 (150.60, 212.69)	179.67 (129.19, 204.25)	
**RIN**					0.039^1^
Median (Q1, Q3)	1.10 (1.00, 1.60)	1.30 (1.00, 1.80)	1.00 (1.00, 1.10)	1.10 (1.00, 1.15)	
**DV200**					< 0.001^1^
Median (Q1, Q3)	41.70 (28.75, 50.25)	32.10 (24.25, 34.35)	24.20 (21.80, 34.40)	25.90 (18.50, 30.10)	
**DV200 above30%**					0.008^2^
No	7 (30.4%)	9 (39.1%)	15 (65.2%)	17 (73.9%)	
Yes	16 (69.6%)	14 (60.9%)	8 (34.8%)	6 (26.1%)	

^1^. Kruskal-Wallis rank sum test

^2^. Pearson’s Chi-squared test

When comparing Method BP and the optimized methods (Method BL), we observed extracts from Method BL had better RNA fragment length for most FFPE samples (13 of 23 vs 16 of 23) (Fig 2B). Overall, there was a marked overlap in the range of RNA purity. All four methods yielded pure RNA extract with similar optimal A_260_/A_280_ ratios (P_260/280_ = 0.265; Kruskal-Wallis). While assessing RNA purity using the A_260_/A_230_ index, purity differed across the four methods (p<0.01) with BP and BL yielding purer material than QP and QE. Method QE yielded significantly higher total RNA concentration compared to the results achieved using other methods (P_RNAyield_<0.05; Kruskal-Wallis). Most extracts from all methods contained relatively degraded RNA, however, extracts from Method BP and Method QE yielded better RIN values ranging from 1.8 to 2.9 (Fig 2C). Fragment length differed across all methods (P_DV200_<0.001; Kruskal-Wallis), with Method BL showing the highest median length and having more extract (16/23 samples) with better fragment length (Fig 2D).

### Influence of RNA extraction method on transcriptomic sequencing

**Fig 3:**
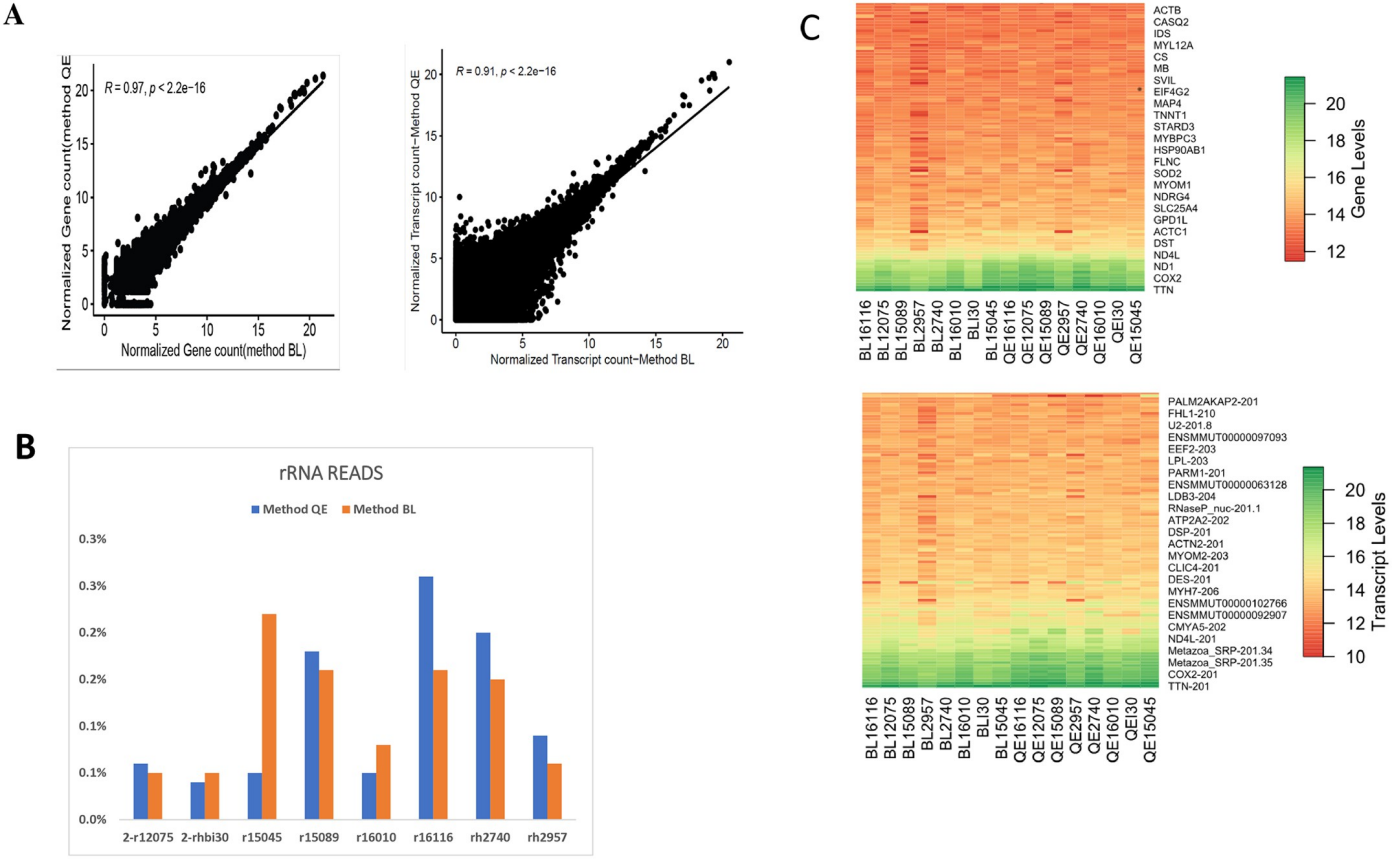
Sequence data comparison between Method BL and Method QE. (A) Correlation plot of normalized gene and transcript count. Data was normalized by log2(TPM+1). Each dot constitutes a gene or transcript. (B) Distribution of Exon, introns, and rRNA mapped reads (C) Heat map of top 25 genes and transcript across extracts from Method QE and Method BL.

As Method QE yielded more extracts with higher RIN values, more RNA yield but less pure RNA extract, and Method BL consistently yielded purer RNA extracts with better fragment length but lower RIN values, we generated libraries from the two methods to evaluate the sequencing performance of Method BL and QE. Sequence data are summarized in Table 3. The average number of reads mapped to the Macaca mulatta (Mmul_10) reference genome averaged 65 million (M) reads (56 M– 87M). Method BL and QE were highly correlated at both gene and transcript levels (ρ_gene-level_ = 0.97, ρ_transcript-level_ = 0.901, Pearson) (Fig 3A). Both methods showed successful removal of most rRNA with < 1% from the total mapped reads 0.1% (0.1–0.3%) (Fig 3B). For direct comparison of gene and transcript count, the data were normalized and transformed as log_2_ values to determine the sensitivity of each Method. At the gene level, the detected number of expressed genes and transcripts was not significantly different in Method QE relative to BL; Method BL, 19,428 genes; Method QE, 19426 genes; Method BL, 40664 transcripts; Method QE, 40687 transcripts (Fig 3C).

**Table 3:** Overview of sequencing characteristics across Method QE and Method BL.

Sample name	Method	Total reads	Total reads to mapping	Total mapped reads	Mapped reads (%)	Intergenic reads (%)	Incompatible paired end	rRNA reads (%)
12075	Additional Ethanol wash step (Method QE)	58,075,340	56,963,596	49,602,531	87.1%	0.5%	2.7%	0.1%
I30	Additional Ethanol wash step (Method QE)	57,680,051	56,690,674	51,791,712	91.4%	0.5%	3.1%	0.0%
15045	Additional Ethanol wash step (Method QE)	73,146,661	71,747,126	66,303,990	92.4%	0.5%	3.2%	0.1%
15089	Additional Ethanol wash step (Method QE)	65,360,592	63,737,851	50,304,992	78.9%	0.5%	2.1%	0.2%
16010	Additional Ethanol wash step (Method QE)	68,586,996	67,403,259	61,641,514	91.5%	0.4%	3.3%	0.1%
16116	Additional Ethanol wash step (Method QE)	62,360,502	61,032,431	49,141,454	80.5%	0.4%	2.1%	0.3%
2740	Additional Ethanol wash step (Method QE)	64,315,815	62,785,413	51,868,787	82.6%	0.4%	2.0%	0.2%
2957	Additional Ethanol wash step (Method QE)	77,138,840	75,208,284	64,952,703	86.4%	0.4%	2.7%	0.1%
12075	Additional Lysis time (Method BL)	59,970,260	58,398,080	50,351,038	88.0%	0.4%	2.1%	0.1%
I30	Additional Lysis time (Method BL)	88,810,749	86,988,897	78,853,990	90.6%	0.4%	2.2%	0.1%
15045	Additional Lysis time (Method BL)	60,565,696	58,285,724	45,489,061	78.0%	0.6%	1.7%	0.2%
15089	Additional Lysis time (Method BL)	62,910,594	61,386,208	55,244,470	90.0%	0.5%	2.0%	0.2%
16010	Additional Lysis time (Method BL)	56,488,938	55,287,689	74,023,242	91.1%	0.5%	2.3%	0.1%
16116	Additional Lysis time (Method BL)	82,231,277	80,857,162	52,538,394	91.5%	0.5%	1.6%	0.2%
2740	Additional Lysis time (Method BL)	64,557,274	62,622,116	51,408,477	100.0%	0.4%	2.1%	0.2%
2957	Additional Lysis time (Method BL)	72,541,306	70,993,146	63,303,135	89.2%	0.5%	2.0%	0.1%

### Differences in cardiac gene expression are protocol-dependent

**Fig 4:**
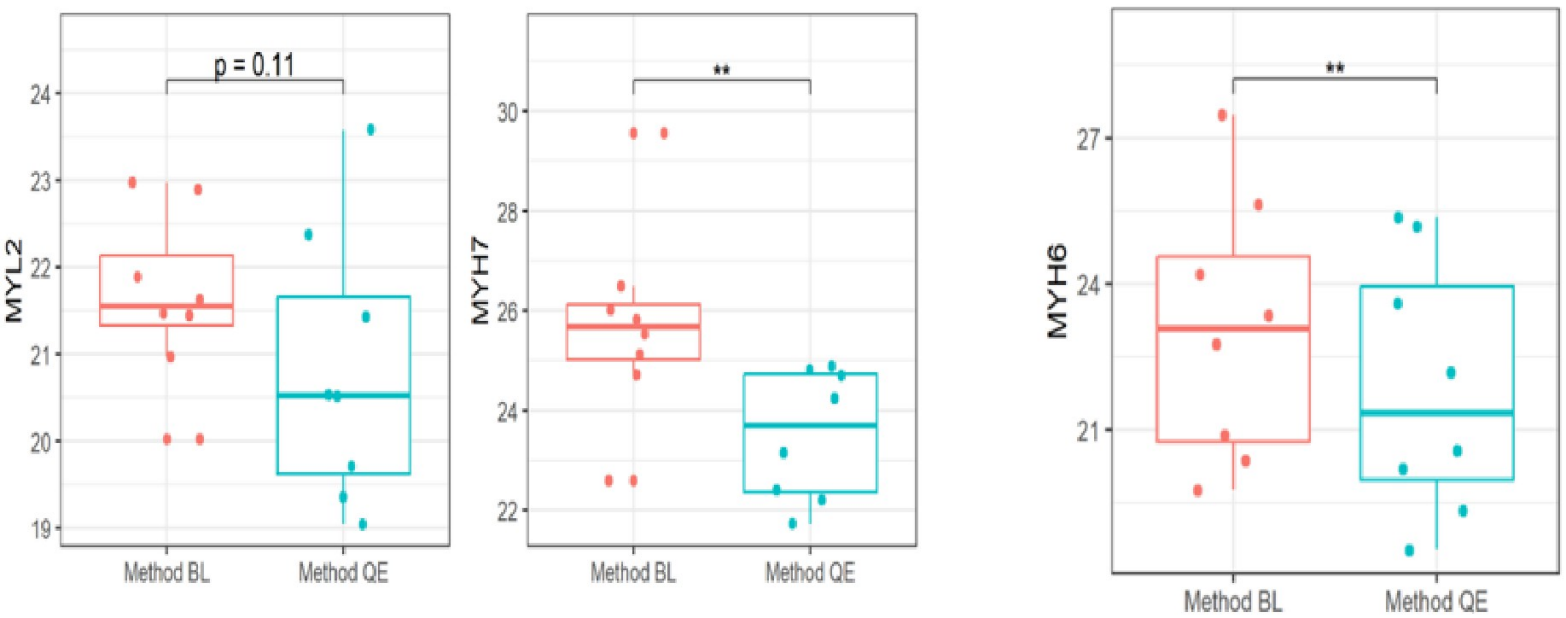
RT-qPCR analysis comparing Method QE and the optimized extraction method (Method BL). (A) Box plots of individual Ct values for genes *MYL2*, *MYH7*, *and MYH6* depict significant differences in gene expression detection. For all groups, an asterisk (*) denotes a significant difference between groups, Paired Wilcoxon Signed Rank Test (*p<0.05, ***p<0.005, ****p<0.001).

We investigated how differences between protocols impacted gene expression values when assayed with the same qPCR protocol and equal RNA quantity. A difference in C_T_−values of MYH6 and MYH7 could be seen for samples extracted with method BL and method QE (median C_T_ 23.09 versus 21.36, *p* = 0.008), (median C_T_ 21.22 versus 20.52, *p* = 0.008) respectively, with samples extracted with the Method BL yielding higher C_T_ values compared to samples extracted with Method QE (Table 4) (Fig 4).

**Table 4:** Summary of qPCR result.

	Method BL (N = 8)	Method QE (N = 8)
**MYH6**		
Median (Q1, Q3)	23.09 (20.76, 24.57)	21.36 (19.98, 23.95)
**MYL2**		
Median (Q1, Q3)	21.55 (21.33, 22.13)	20.52 (19.62, 21.66)
**MYH7**		
Median (Q1, Q3)	25.67 (25.02, 26.13)	23.69 (22.36, 24.72)

## Discussion

The molecular landscape comprising the transcriptome, proteome, and genome is crucial to understanding the biological complexity of both normal and aberrant mammalian processes. Therefore, precise, and efficient molecular techniques are required to unravel these complexities. We evaluated different methods of extracting RNA from archived FFPE cardiac tissue from non-human primates and assessed the impact of RNA quality metrics on gene expression and transcriptome sequencing. In identifying several significant contributors to quality transcriptome sequencing outcomes, we demonstrate that high-quality RNA extraction from cardiac tissue is contingent on additional RNA extraction steps much more than predesigned commercially available kits chemistry.

In examining the hypothesis that additional steps beyond the manufacturer’s protocol could improve RNA yield and integrity of FFPE-derived samples and subsequently improve sequence outcomes, we observed that the optimized protocol (Method QE) resulted in a higher number of samples with DV200 above 30% compared to Method QP (8 of 23 vs. 6 of 23). This implies that fragment length may be slightly impacted by an extra ethanol wash step following deparaffinization. In comparing the CELL DATA RNA FFPE extraction kit to the optimized method, we found that extending the protease treatment to 24 hours (Method BL), rather than the 2 hours recommended by the manufacturer (Method BP), significantly improved RNA yield and resulted in better RNA fragment lengths in most samples extracted with Method BL compared to Method BP (16 of 23 vs. 13 of 23).

This supports the notion that an incubation temperature of 72°C may be optimal for effective protease activation during extraction [24]. However, archived FFPE material still requires modifications in protease incubation time to effectively overcome cross-linking. For evidence of this, previous results from Boos and colleagues show that an RNA protocol requiring a brief proteinase K incubation period produced an extract that, when amplified by qPCR, did not amplify the smallest housekeeping genes (48 bp and 64 bp), but the extract from a longer incubation period revealed amplicons that were 170 bp–298 bp long [13]. We propose that combining Method BP with this adaptation to the lysis step provides a reproducible approach, demonstrating that Method BL is a suitable tool for obtaining RNA with improved fragment length. However, a significant limitation of Method BL for routine clinical diagnostics is its extended incubation time of 25 hours and 30 minutes, resulting in a total turnaround time of 27 hours and 46 minutes. Therefore, we recommend this protocol only when time constraints are not a priority.

All methods produced a sufficient yield of RNA required for our sequencing protocol. The highest RNA yield was obtained with method QE, however, in terms of DV 200 values above 30%, our results showed the highest DV200 values for method BL in all samples compared with those for methods QP, QE, and BP. Previous research has equally reported significant differences in DV200 values among various extraction methods [25]. When assessing RNA purity using the A260/230 absorbance ratios, our data also showed that Method QE and QP had significantly lower 260/230 nm absorbance ratios compared with methods BL and BP, indicating the presence of contamination with organic solvent that absorbs light strongly at or near 230 and 260 nm which possibly could have affected the distribution of the size of the final extracted RNA molecules.

Data generated in our current study indicate that nucleic acid quality in FFPE tissue specimens is adversely affected by formalin fixation [11, 14] as all methods deployed for FFPE extraction consistently yielded RNA of low RIN score. This raises the question of the usefulness of RIN value as a quality metric for FFPE tissue samples. The RNA integrity number (RIN) is used to assess RNA quality through an algorithm that considers rRNA peak ratios, separation, and the presence or absence of degradation products [26]. However, multiple studies have shown that RIN values are not a reliable indicator of RNA quality in FFPE samples, nor do they accurately predict performance in downstream molecular analyses [25, 27, 28]. Our current evaluation of RIN scores in FFPE samples supports these previous observations.

Our RNA sequencing results demonstrate that Method QE and Method BL achieved similar performance deploying similar sequencing protocols, based on the evaluation of mapping variables, transcript counts, gene counts, and rRNA contamination. Although the 260/230 nm absorbance ratios of the extract from Method QE were significantly lower and relatively less pure than those of Method BL, this did not negatively influence the sequencing outcome. By comparing transcriptome sequences of extract from Method QE and BL, we demonstrated that RNA extracts with optimal purity and low RIN value but fragment length size above 30% (Method BL) and RNA extracts with high RNA yield, low RIN value but fragment length size above 30% can generate high-quality transcriptome sequences (Method QE). The suggests that for generating high-quality transcriptome sequences, the RNA fragment length (above 30%) may be more critical than the RNA Integrity Number (RIN). Both methods, despite differences in their strengths—purity for Method BL and yield for Method QE—were effective, as long as the fragment length threshold was met. This finding emphasizes the adaptability of RNA sequencing protocols to samples with varying RNA quality characteristics. Recently, Illumina developed the DV200 to assess RNA quality in FFPE samples for successful library preparation for next‐generation sequencing. The broader scope of the DV_200_ assay makes it a more meaningful predictor of FFPE sample performance for whole transcriptome analyses than RIN value, RNA yield, and RNA purity assessment with A260/230 [29, 30].

To highlight the importance of RNA quality on gene expression detection, our data shows significant differences in cardiac gene expression as RNA extract from method QE had low CT/higher gene expression than extract from Method BL. This collaborates with the findings of earlier studies [31–33] where RNA quality has a measurable impact on gene expression results. Overall, our qRT-PCR results suggest that despite widespread changes affecting the electrophoretic trace of a sample, the qRT-PCR technique is sensitive to detect more/less abundantly expressed genes and the integrity of individual transcripts remains stable for both abundantly expressed and less abundantly expressed genes.

While we are aware that our relatively small sample size could have led to the overestimation of our result, we anticipate that these findings will be of interest to investigators who routinely quantify gene expression and transcriptomes from FFPE tissues and require a more reproducible method beyond conventional methods. Results from our data will continue to build the body of knowledge on the fitness of FFPE cardiac tissues for various transcriptomics platforms, improve biobanking efforts, and lend support for standardization efforts to improve the quality and reproducibility of molecular data ultimately.

## Supporting information

S1 Table Gene-specific primer sequence for RT- qPCR. (DOCX)

## Abbreviations

*C_T_*: Cycle threshold
CVD: Cardiovascular disease
*FFPE*: Formalin-fixed paraffin-embedded
*NGS*: Next-generation sequencing
*RIN*: RNA Integrity Number
