# Supplementary material for: Optimizing RNA extraction methods for high-throughput transcriptome sequencing of formalin-fixed paraffin-embedded cardiac tissue specimens
Source: PLoS One. 2024 Dec 26;19(12):e0315098. doi: 10.1371/journal.pone.0315098 (PMC11670944; doi:10.1371/journal.pone.0315098)
Supplement: S1 Table — (DOCX) [file pone.0315098.s001.docx]

**Supplementary Data**

**Table S1: Gene-specific primer sequence for RT- qPCR**

| **Gene** | **Primers** | **Sequence** |
| --- | --- | --- |
| ***MYL2*** | Forward | 5`-CAGAACAGGGATGGCTTCAT-3` |
|  | Reverse | 5`-CGGAGCCTCCTTGATCATTT-3` |
| ***MYH6*** | Forward | 5`-GCAGACTGAGAATGGAGAGTTG-3` |
|  | Reverse | 5`-GCTCCTGGGAGCAGCGGTA-3` |
| ***MYH7*** | Forward | 5`-GAGACTGTAGTGGCCTTGTATC-3` |
|  | Reverse | 5`-CTTTCTTGGCCTTGCCTTTG-3` |
